# Genetic investigation of biological materials from patients after stem cell transplantation based on autosomal as well as Y-chromosomal markers

**DOI:** 10.1007/s00414-012-0771-x

**Published:** 2012-09-28

**Authors:** Renata Jacewicz, Krzysztof Lewandowski, Joanna Rupa-Matysek, Maciej Jedrzejczyk, Mieczysław Komarnicki, Jarosław Berent

**Affiliations:** 1 Forensic Genetics Laboratory, Department of Forensic Medicine, Medical University of Lodz, Sedziowska 18 a, 91-304 Lodz, Poland; 2 Department of Hematology, Poznan University of Medical Sciences, Szamarzewskiego 84, Poznań, 60-569 Poland; 3 Department of Forensic Court and Insurance Certification, Medical University of Lodz, Sedziowska 18 a, 91-304 Lodz, Poland

**Keywords:** Stem cell transplantation, Forensic genetics, DNA analysis, Y chromosome markers

## Abstract

The authors presented the results of DNA polymorphism investigation of blood, buccal swabs and hair follicles originating from patients after allogeneic hematopoietic stem cell transplantation. The real-time and multiplex assays based on polymerase chain reaction within the range of autosomal as well as Y-chromosomal markers were applied to assess the possible dangers arising from investigation of these materials in forensic genetics. The results revealed that not only post-transplant blood and buccal swab, but also recipient hair, up to now regarded as devoid of any donor’s cells, do not constitute entirely safe material for forensic purposes. Their analysis can lead to the false identification of gender or male haplotype. The investigation of sex-determining region Y and Y-chromosome short tandem repeats performed in female recipients with male donors resulted in the designation of donor’s DNA in hair cells as well as in blood and buccal swabs. Therefore, biological stains gathered from crime scenes should not be analysed exclusively based on the investigation of male-specific markers.

**Electronic supplementary material:**

The online version of this article (doi:10.1007/s00414-012-0771-x) contains supplementary material, which is available to authorized users.

## Introduction

At the core of the research in forensic genetics is the assumption that all cells creating a single individual have the same DNA profile. This assumption enables the personal identification and kinship analysis based on the extraction of DNA from human remains or different human materials [1]. It allows the linking of offenders to crime scenes by matching DNA samples secured from a wide range of biological stains or taken from suspects to genetic profiles that have been stored in the national DNA databases [2].

However, there are some reports proving that various tissues may differ genetically and do not always have the DNA profile of the person from whom they originate. This can be the result of mutation [3] or congenital chimerism of twins [4], resulting from the partial exchange of hematopoietic stem cells during the foetal life. It is also possible when the person, from whom the biological stain or tissues come from, underwent allogeneic hematopietic stem cell transplantation (allo-HSCT) in the past [5]. Numerous reports document the ability of bone marrow stem cells to transform into cells other than hematopoietic ones as well as their ability to move within different tissues and organs [6–8]. This can lead to the existence of heterogeneous or diverse DNA material extracted from various tissues of the recipient, which would constitute a significant threat in forensic genetic investigations.

Therefore, the aim of the work was the DNA polymorphism analysis of blood, buccal swabs with epithelial cells as well as hair follicles originating from patients after allo-HSCT, and evaluation of any dangers arising from the investigation of these materials both in the identification and kinship analysis.

## Material and methods

Thirty-two patients, who had undergone successful allogeneic hematopoietic stem cell transplantation between 32 and 2,764 days previously, were recruited from the Department of Haematology at the Poznan University of Medical Sciences in Poland. Seven patients had unrelated donors, 24 patients had sibling donors and one patient had a parent donor. Sixteen women recipients had received allo-HSCT from male donors. The Bioethics Committees of Medical Universities of Lodz and Poznan gave their consent to the research owing to decisions no. RNN/74/08/KE and no. 270/08, respectively. The informed consent for the investigation was received from all the patients. The characteristics of patients included in analyses are shown in Electronic supplementary material (ESM) Table 1.

The post-transplant peripheral blood and buccal swabs from every recipient as well as the reference material, i.e. pre-transplant recipient and donor blood samples were collected in duplicate and frozen until DNA extraction. As well as this, four to 10 post-transplant hairs with roots from each recipient were plucked and kept at room temperature before the duplicate DNA extraction. The genomic DNA from all the investigated materials was isolated by the Sherlock AX kit (A&A Biotechnology). The total concentration of each DNA sample was evaluated with a Qubit® fluorometer and Quant-iT™ dsDNA HS kit (Invitrogen). The detection of the sex-determining region Y (SRY) and the male DNA quantity was performed with Quantifiler® Duo DNA Quantification Kit in 7500 Real-Time polymerase chain reaction (PCR) System with HID Analysis Software v.1.0. The co-amplification with 5-dye detection of the 15 autosomal unlinked loci and gender-determining amelogenin was performed based on the AmpFlSTR® Identifiler™ PCR Amplification Kit according to the manufacturer’s protocol (Applied Biosystems). Additionally, in male to female samples and in male to unrelated male samples co-amplification with 5-dye detection of the 17 STR loci along the Y-chromosome was conducted with the use of the AmpFlSTR Yfiler™ PCR Amplification Kit (Applied Biosystems). Allele detection was performed with GeneMapper® ID-X Software v.1.2. using LIZ 600 v.2 size standard with reference allelic ladder in 3500 Genetic Analyzer (Applied Biosystems). Donor chimerism admixture was assessed according to Nollet et al. [9].

## Results and discussion

The assumption that all the cells in the human body have the same DNA profile constitutes the basis for comparative analysis in forensic genetics. This conviction allows genetic profiles obtained from different tissues of particular persons to be compared in regard to the kinship analysis as well as personal identification [1]. It gives the possibility to match a DNA profile recovered from a blood stain or hair taken from a crime scene to a DNA profile that has been stored in the DNA database after its determination in the buccal swab taken from a suspect [2].

Nevertheless in special cases, this assumption can be wrong. It can take place when tissues of recipients after allogeneic stem cell transplantation are investigated [5, 6]. Adult stem cells can cross lineage barriers and differentiate into cells outside their own tissue [10–13]. Experimental and clinical studies have identified the donor’s cells in different tissues of the recipients’ body [14–17].

The present work evaluated the dangers arising from the investigation of blood and buccal swabs with epithelial cells as well as hair follicles, which are among the most commonly used materials in forensic genetics, taken from patients after marrow cell transplantation.

The analysis of autosomal STR markers performed in the investigated materials revealed the full donor’s DNA profile in most of the blood recipient post-transplant samples and the mixture (11–67 %) of the recipient’s and donor’s pattern in the majority of buccal swabs. The remaining recipients revealed a mixed profile (63–75 %) in blood and a full autological profile in buccal swabs (see ESM Table 1). The results of polymorphism DNA investigation received for representative female patient no. 11, who underwent successful allogeneic hematopoietic stem cell transplantation, is presented in ESM Fig. 1. It detailed the results of CSF1PO locus—one out of 15 amplified autosomal STR markers—detected subsequently in post-transplant recipient’s materials: blood, epithelial buccal swab and hair follicles as well as in pre-transplant donor’s and recipient’s samples.

These results are in agreement with earlier reports [5, 16–19]. The donor-derived cells were detected not only in blood and buccal epithelial cells but also in fingernails, which regenerate continuously throughout life and have the same ectodermal origin as the hair [5, 15, 20]. In spite of this, the hair follicles of all of the 32 investigated recipients after successful allo-HSCT had a solely autologous profile within autosomal DNA without the detection of any donor-origin cells (ESM Fig. 1). Some scientific efforts have been undertaken earlier to reveal the donor-derived cells in recipient hair. Dauber et al. [5], Rovo et al. [21], Hong et al. [17], Zhou et al. [18] and, more recently, Berger et al. [19], applying the autosomal STR-PCR analysis as the only investigation method, documented a complete lack of susceptibility for conversion to donor type in hair from recipients of allo-HSCT. These results convince that only hair follicles, and not fingernails or buccal swabs, remain completely of the recipient’s origin. It was concluded that hair follicles are the only reliable source of biological material for forensic investigation.

The presented research, similar to the previously performed investigations [22], proved the aforementioned conviction to be false. The key to invalidating this belief was the application of highly sensitive and male-specific methods enabling the selective amplification of Y-chromosome markers. A similar approach based on sex mismatched allografts was applied by Tran et al. [23].

**Fig. 1:**
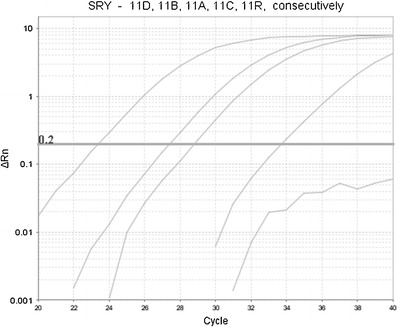
Amplification plot from a male specific real-time PCR assay with SRY sequence (Quantifiler® Duo DNA Quantification Kit) detected consecutively in the donor’s sample (*D*), post-transplant recipient’s materials: buccal swab (*B*), blood (*A*), hair follicles (*C*) and pre-transplant recipient’s control (*R*)

In the samples taken from 16 women who received engraftment from a male donor, we performed TaqMan probe-based real-time PCR male specific assay as a previous step to the Y-chromosome short tandem repeat (Y-STR) multiplex analysis. The amplification plot from the SRY detected in post-transplant materials from the representative female (no. 11), who received allo-HSCT from her brother as well as in the donor’s sample and pre-transplant recipient’s control is presented in Fig. 1.

The real-time PCR assay revealed in post-transplant samples of blood and buccal swabs, obtained from all 16 investigated women, donor-derived male DNA in the range of 1.56–52.28 and 0.93–35.36 ng/μl, respectively. In the hair follicles of 12 out of 16 female recipients after male engraftment, the sequence of SRY gene was revealed, which corresponds with male DNA ranging from 0.01 to 0.19 ng/μl found in the total DNA amount of 0,53–20,31 ng/μl. Therefore, every one male cell of donor origin detected in investigated women hair follicles coincided with several dozen, a few hundred or even a few thousand female ones.

The Y-STR study performed as the next step in the analysis of Y-chromosome markers revealed the male haplotype of donor origin in all 16 post-transplant blood and buccal swabs samples and unexpectedly also in post-transplant hair follicles taken from 14 out of 16 examined women (87.5 %) after allo-HSCT. A full donor Y-haplotape with 17 loci was revealed in the hair follicles of 10 female recipients, while in the four of them a partial donor Y-haplotape with three to nine loci was detected. Only in the hair follicles of two remaining women (12.5 %), where SRY gene was undetected, none of 17 Y-STR markers were revealed. The Y-STR haplotype, received for representative female recipient no. 11 is presented in ESM Fig. 2. It detailed DNA alleles within three (DYS439, DYS635, DYS392) out of 17 investigated loci, detected in her blood, epithelial buccal swabs, hair follicles and pre-transplant control and as well as in the sample of the male donor.

The detection of donor-derived DNA fractions based on the analysis of SRY and Y-STR markers and the lack of any donor fractions in parallel performed analysis of autosomal markers in hair derived from females, who had undergone transplantation with male hematopoietic stem cells, has a relatively simple explanation. The overwhelming quantities of the female DNA mixed with the male DNA caused the preferential amplification of the female fraction and the inhibition of the amplification of the male one [24]. This is a well-known fact in forensic genetics, e.g. in samples collected after sexual assaults [25]. A very small amount of the donor’s origin cells in the recipient’s hair at the level of 0.01–0.0001 did not allow for the appearance of the minor component of the mixture. On the other hand, the use of highly sensitive markers allowing for selective amplification of male donor cells revealed SRY region and donor’s Y-STR haplotype not only in post-transplant blood and buccal swabs, but also in the hair follicles of the recipients.

What is important, we found no SRY sequence and no Y-STR markers in any of the pre-transplant control samples taken from the investigated women recipients who had received allo-HSCT from male donors just as was the case with the representative female recipient presented in Fig. 1 and ESM Fig. 2. Thus, we ruled out the hypothesis that the observed donor’s fraction in the hair of the females was the result of prenatal male microchimerism.

## Conclusion

To sum up, our investigation confirmed that hair follicles as well as blood and buccal swabs are not an entirely safe material for forensic purposes. Their analysis based on the autosomal DNA revealed 100 % of the recipient’s profile. However, the analysis based on Y-chromosome markers performed in female recipients with male engraftment, resulted in the designation of the SRY gene and Y-STR haplotype of donor origin in hair cells as well as in blood and buccal swabs. Therefore, the biological stains gathered from crime scenes should not be analysed exclusively based on the analysis of Y-chromosome markers.

## Electronic supplementary material

Below is the link to the electronic supplementary material.

ESM 1 233 kb
