# Supplementary material for: Genetic investigation of biological materials from patients after stem cell transplantation based on autosomal as well as Y-chromosomal markers
Source: Int J Legal Med. 2012 Sep 28;127(2):359–62. doi: 10.1007/s00414-012-0771-x (PMC3578715; doi:10.1007/s00414-012-0771-x)
Supplement: Supplementary file 1 — 233 kb [file 414_2012_771_MOESM1_ESM.doc]

**Supplementary material**

**Genetic investigation of biological materials from patients after stem cell transplantation based on autosomal as well as Y-chromosomal markers.**

Renata Jacewicz, Krzysztof Lewandowski, Joanna Rupa-Matysek, Maciej Jedrzejczyk, Mieczysław Komarnicki, Jarosław Berent

R.Jacewicz, J.Berent - Forensic Genetics Laboratory, Department of Forensic Medicine, Medical University of Lodz, Sedziowska 18 a, 91-304 Lodz, Poland

K. Lewandowski, J. Rupa-Matysek, M. Komarnicki

Department of Hematology, Poznan University of Medical Sciences, Szamarzewskiego 84, Poznań 60-569, Poland

M. Jedrzejczyk - Department of Forensic Court and Insurance Certification, Medical University of Lodz, Sedziowska 18 a, 91-304 Lodz, Poland

**corresponding author:**

Renata Jacewicz, Ph.D.

Head of Forensic Genetics Laboratory
Department of Forensic Medicine
Medical University of Łódź
ul. Sedziowska 18a, 91-304 Lodz
Poland
tel. (+48) (42) 654-45-36, 654-53-88, 665-26-43
fax (+48) (42) 654-42-93
mail [r.jacewicz@post.pl](https://poczta.home.pl/mail/write?to=r.jacewicz@post.pl)
<http://www.umed.lodz.pl/ou/zms>/

**Supplementary Table 1**

Characteristics of patients after allo-HSCT (n=32) and chimerism studies in their posttransplant materials: blood, buccal swab and hair follicle in a range of autosomal as well as Y-chromosomal markers

| **Recipient (years)** - range  median/average | 22-59  36/40 |
| --- | --- |
| **Time post allo-HSCT (days)** - range  median/average | 32-2764  588/945 |
| **Diagnosis**  AML/CML/ALL/AA/HD/Lyphoma/CLL | 15/7/3/2/2/2/1 |
| **Conditioning regiments**  MA/NM | 16/16 |
| **Source of hematopoietic stem cells**  PB/BM/PB&BM | 25/5/2 |
| **Related/Unrelated Donor** | 25/7 |
| **Donor-Recipient Gender**  M-F/M-M/F-F/F-M | 16/4/7/5 |
| **Patients with chimerism - autosomal DNA**  Blood (donor’s/mixed/recipient’s)  Buccal swab (donor’s/mixed*/recipient’s)  Hair follicles (donor’s/mixed*/recipient’s) | 30/2/0  0/18/14  0/0/32 |
| **Patients with chimerism - chromosome Y****  Blood/buccal swab/hair follicles(undetected) | 16/16/14(2) |

AML - acute myeloid leukaemia, CML - chronic myeloid leukaemia, ALL - acute lymphoblastic leukaemia, AA – aplastic anemia, HD - Hodgkin’s disease, CLL – chronic lymphocytic leukaemia, MA-myeloablative, NM – non-myeloablative,
PB - peripheral blood, BM - bone marrow, M-male, F-Female

* mixed chimerism ranging from 63 to 75% in blood and from 11 to 67% in buccal swabs

****** exclusively donor’s type chimerism as a result of selective amplification of male Y-STR sequencies in female recipients

**Supplementary Fig. 1** Polymorphism of CSF1PO locus out of 15 autosomal STR loci after AmpFlSTR® Identifiler™ kit amplification of the samples from representative patient No.11. Profiles shown from top to bottom: post-transplant recipient’s: blood (A), buccal swab (B) and hair follicles (C), donor’s sample (D) and pre-transplant recipient’s control (R). The genotype is shown with the allele number displayed underneath each peak

**Supplementary Fig. 2** Detection of three out of 17 Y-STR loci after AmpFlSTR Yfiler™ kit amplification of the samples from representative patient No. 11 – a female receiving allo-HSCT from a male. Profiles shown from top to bottom: post-transplant recipient’s: blood (A), buccal swab (B) hair follicle (C), donor’s sample (D) and pre-transplant recipient’s control (R). The haplotype is shown with the allele number displayed underneath each peak
